# Sirtuins and redox signaling interplay in neurogenesis, neurodegenerative diseases, and neural cell reprogramming

**DOI:** 10.3389/fnins.2023.1073689

**Published:** 2023-02-01

**Authors:** Elisabetta Mormone, Eugenio Luigi Iorio, Lucrezia Abate, Carlo Rodolfo

**Affiliations:** ^1^Unitá Produttiva per Terapie Avanzate, Fondazione IRCCS Casa Sollievo della Sofferenza, San Giovanni Rotondo, Italy; ^2^International Observatory of Oxidative Stress, Salerno, Italy; ^3^Department of Biology, University of Rome Tor Vergata, Rome, Italy; ^4^Department of Paediatric Onco-Haematology and Cell and Gene Therapy, IRCCS Bambino Gesù Children’s Hospital, Rome, Italy

**Keywords:** NSCs, neurogenesis, mitochondria, reactive oxygen species (ROS), Sirtuins (SIRTs), neurodegenerative diseases (NDs), 8-oxoguanine DNA glycosylase (OGG1)

## Abstract

Since the discovery of Neural Stem Cells (NSCs) there are still mechanism to be clarified, such as the role of mitochondrial metabolism in the regulation of endogenous adult neurogenesis and its implication in neurodegeneration. Although stem cells require glycolysis to maintain their stemness, they can perform oxidative phosphorylation and it is becoming more and more evident that mitochondria are central players, not only for ATP production but also for neuronal differentiation’s steps regulation, through their ability to handle cellular redox state, intracellular signaling, epigenetic state of the cell, as well as the gut microbiota-brain axis, upon dietary influences. In this scenario, the 8-oxoguanine DNA glycosylase (OGG1) repair system would link mitochondrial DNA integrity to the modulation of neural differentiation. On the other side, there is an increasing interest in NSCs generation, from induced pluripotent stem cells, as a clinical model for neurodegenerative diseases (NDs), although this methodology still presents several drawbacks, mainly related to the reprogramming process. Indeed, high levels of reactive oxygen species (ROS), associated with telomere shortening, genomic instability, and defective mitochondrial dynamics, lead to pluripotency limitation and reprogramming efficiency’s reduction. Moreover, while a physiological or moderate ROS increase serves as a signaling mechanism, to activate differentiation and suppress self-renewal, excessive oxidative stress is a common feature of NDs and aging. This ROS-dependent regulatory effect might be modulated by newly identified ROS suppressors, including the NAD^+^-dependent deacetylase enzymes family called Sirtuins (SIRTs). Recently, the importance of subcellular localization of NAD synthesis has been coupled to different roles for NAD in chromatin stability, DNA repair, circadian rhythms, and longevity. SIRTs have been described as involved in the control of both telomere’s chromatin state and expression of nuclear gene involved in the regulation of mitochondrial gene expression, as well as in several NDs and aging. SIRTs are ubiquitously expressed in the mammalian brain, where they play important roles. In this review we summarize the current knowledge on how SIRTs-dependent modulation of mitochondrial metabolism could impact on neurogenesis and neurodegeneration, focusing mainly on ROS function and their role in SIRTs-mediated cell reprogramming and telomere protection.

## 1. Introduction

Adult neurogenesis (AN) can be defined as “the birth and development of new neurons in adulthood” (Just et al., 2022). The discovery of the existence of neural progenitor cells (NPCs) in the mice brain, paved the way to the identification of specific brain areas, containing cells with a neurogenic potential, termed neural stem cells (NSCs) (Reynolds and Weiss, 1992). A better understanding of the mechanisms controlling adult neurogenesis could be the key for the treatment of a wide range of neurodegenerative, neuropsychiatric, and metabolic disease (Just et al., 2022). In the adult mammalian brain, NSCs mainly reside in the subventricular zone (SVZ) located in the wall of the lateral ventricles, and the sub-granular zone of the dentate gyrus (DG), in the hippocampus (Llorente et al., 2022). NSCs are capable to self-renew and, when needed, to differentiate into the diverse cell types of the nervous system. In the last years, extensive research focused on the understanding and the possible manipulation of the molecular pathways preserving NSCs function, as well as on their usage in transplantation, to restore cognitive and behavioral deficits, in preclinical models of neurodegenerative diseases (NDs). Although many progresses have been made in the field, some aspects still need to be clarified, such as the role of mitochondrial metabolism in the regulation of endogenous adult neurogenesis and neurodegeneration. In addition, considering the limited migratory capacity and the low availability of NSCs for allogenic transplantation, the interest is moving toward the definition of a suitable protocol for generating them from induced pluripotent stem cells (iPSCs). Cell reprogramming could be a powerful technique for the regenerative medicine filed, although still characterized by several drawbacks generated during the reprogramming process, such as the telomere shortening. Moreover, the transcription factors used in somatic cells reprogramming protocols may alter the genomic contents, leading to genetic mutations (Chen et al., 2019), as well as to impact on mitochondrial dynamics, resulting in excessive mitochondrial fission and ROS production, thus greatly limiting pluripotency and reprogramming efficiency. Furthermore, excessive ROS production might induce apoptosis in the transplanted cells (Sart et al., 2014), although recent studies challenged this dogma by demonstrating a physiological role for ROS in the regulation of stem cell fate decision (Sart et al., 2015). Indeed, iPSCs proliferation and differentiation are actively controlled by mild levels of ROS (e.g., 1.8-fold the normal level) (Lee et al., 2010). All these aspects, including genomic instability and impaired mitochondrial dynamics, should be addressed to exploit cell reprograming for clinical research as well as to comply efficiency and safety concerns.

Recent studies showed the Sirtuins (SIRTs) protein family as involved in the modulation of a variety of cellular processes, associated with antioxidant (AOX) and redox signaling. In detail, SIRT1, SIRT3, and SIRT5 protect the cell from ROS; SIRT2, SIRT6, and SIRT7 modulate key oxidative stress genes and mechanisms; whereas SIRT4 induces ROS production and performs antioxidative roles, as well (Singh et al., 2018). In addition, SIRTs are involved in the control of genomic instability (Shin et al., 2018). Despite the remarkable number of works about the role of metabolic switch in neurogenesis regulation, it is not completely understood how SIRTs might regulate neurogenesis through mitochondria metabolism (Tay et al., 2021), particularly through ROS and NAD system, as well as their role in telomere length and genomic stability maintenance, during cell reprogramming.

The purpose of this review is to summarize the current knowledge about the emerging role of SIRTs, with a focus on SIRT1, SIRT2, and SIRT3, as regulators of neurogenesis through metabolic modulation and ROS signaling, in NDs and aging as well as their role in the cell reprogramming. We think that genetic, hormonal, and drug manipulation of NSCs mitochondria, may be useful to prolong NSCs longevity and stability prior to their clinical usage, or even to improve their endogenous function. Therefore, a better understanding of the molecular mechanisms underlying neurogenesis and cellular reprogramming may be of help to identify new potential therapeutic targets.

## 2. Mitochondria alterations in neurodegenerative diseases

Mitochondria are essential organelles for cell’s life and death, as they not only provide ATP but are central in the modulation of several cellular pathways, from Ca^2+^ signaling to apoptosis. Alteration of mitochondrial functions is a common feature of both apoptosis and autophagy. During apoptosis, mitochondria integrate intrinsic and extrinsic death signals, with the loss of mitochondrial membrane potential (MMP) and the permeability transition pore considered as a latest executioner event associated with the release, from the mitochondrial intermembrane space, of cofactors required for caspases’ activation (Wang and Youle, 2009). MMP loss could also be a signal for the induction of mitochondrial fission and subsequent elimination of the damaged organelles, through a specialized form of autophagy, termed mitophagy (Mormone et al., 2006; Li et al., 2021). Functional versatility of mitochondria is matched by a complex morphology as they not only display a complex ultrastructure, but also appear interconnected and networked (Anesti and Scorrano, 2006). In humans, the shape of both individual mitochondria and the mitochondrial network depends upon fission and fusion processes, which are tightly regulated by the so called “mitochondria-shaping” proteins (Dhingra and Kirshenbaum, 2014), able to promote fission (Drp1, Fis1), fusion (Mfn1/2, Opa1), and transport (Miro, Milton) of the organelles (Detmer and Chan, 2007). The mitochondrial network is an extremely dynamic structure subjected to continuous changes during cell-cycle progression and cell division. The importance of mitochondrial dynamics is further substantiated by the observation that mutations in mitochondria-shaping proteins can result into NDs (Panchal and Tiwari, 2019), like autosomal-dominant optic atrophy and Charcot-Marie-Tooth (Carelli et al., 2009). In addition, mitochondrial dysfunction, caused by excessive oxidative stress, depletion of cellular energy levels, and defective protein production, associate with dopaminergic neurons loss in Parkinson’s disease (PD). Sporadic PD etiopathogenesis is complex, comprising both environmental and genetic factors, able to affect mitochondrial life cycle, bioenergetic capacity, quality control, dynamic changes (fusion and fission), subcellular distribution, as well as cell death pathways regulation (Fukae et al., 2007; Prasuhn et al., 2020). In addition, in a mouse model of Alzheimer’s disease (AD), it has been reported that the expression of the mitochondrial transport and dynamics regulator Miro2, which is degraded through PINK1/Parkin-dependent mitophagy (Saotome et al., 2008; Shlevkov et al., 2016), was decreased in Nestin-positive cells of the hippocampus (Woo et al., 2021). Indeed, in cultured adult hippocampus-derived NSCs of normal mice, Miro2 downregulation results into excessive mitochondrial fission, increased ROS production, and autophagic cell death, rescued by Miro2 over-expression and pharmaceutical inhibition of Drp1 activity (Mdivi-1) (Woo et al., 2021). In addition, both α-Synuclein (α-SYN) and amyloid-β (Aβ), responsible for neurotoxic protein aggregates accumulation in PD and AD, impair mitochondrial respiration (Manczak et al., 2006; Reeve et al., 2015), suggesting that mitochondrial defects also contributed to abnormal adult hippocampal neurogenesis (AHN) (Zhang et al., 2019; Amber et al., 2020). Taken together, this evidence supports the idea that mitochondrial-associated abnormalities of adult hippocampal neurogenesis, contribute to cognitive and psychiatric disturbances in neurodegenerative illnesses. In Huntington’s disease (HD), the degenerative process relies on both the acquisition of toxic function by mutated huntingtin as well as on the loss of protection exerted by the wild type protein, leading to diverse cellular alterations. Indeed, mitochondria impairment and increased oxidative stress result into cell death induction, by apoptosis and/or autophagy (Mormone et al., 2006; Duan et al., 2014; Panchal and Tiwari, 2019). *In vitro* and *in vivo* observations for Amyotrophic Lateral Sclerosis (ALS), suggested that mutation of genes associated with the disease (SOD1, TDP-43, FUS, and TAF15), can alter mitochondrial dynamics and induce oxidative stress (Panchal and Tiwari, 2019; Kodavati et al., 2020), coupled to the nuclear accumulation of the nuclear factor erythroid 2-related factor 2 (NRF2), a master regulator of detoxification, AOX, and anti-inflammatory mechanisms (Obrador et al., 2020).

Multiple sclerosis (MS) is a chronic demyelinating disease of complex etiology affecting the CNS, where oligodendrocytes act as myelination cells, in which a role for mitochondrial dysfunction preceding the axonal damage has been suggested (Heidker et al., 2017; Wentling et al., 2019). Indeed, it has been suggested that inflammatory demyelination could result into neurodegeneration through different mechanisms including energy depletion, due to mitochondrial dysfunction and/or hypoxia related processes, activated microglia, oxidative stress, activated astrocytes, iron accumulation, Wallerian degeneration, and apoptosis. Hence, while the primary therapeutic approach is still directed against the immune system, new experimental protocols, aiming to lessen or delay MS progression, are focused on neuronal and glial metabolism support and/or remyelination promotion (Heidker et al., 2017).

Mitochondrial dependent generation of ROS proved to be a common feature of NDs (Liu et al., 2017) and related to neuronal injury and pathological progression (Ismail et al., 2020). A better understanding of the molecular pathways controlled by mitochondrial metabolism, through oxidative stress, would be useful to design new therapeutic approaches targeting specific proteins or molecules.

## 3. Mitochondrial and ROS metabolism

### 3.1. Metabolic regulation of neural stem cell fate

Energy requirements of brain cells are quite diverse, with neurons relying on mitochondrial-based oxidative phosphorylation (OXPHOS) and glia cells mostly on glycolysis (Lopez-Fabuel et al., 2016). SGZ and SVZ resident NSCs, preferentially rely on aerobic glycolysis, while their more differentiated progeny generates ATP mainly by OXPHOS (Rafalski et al., 2012; Zheng et al., 2016; Lorenz and Prigione, 2017). Proteomic analysis of cultured NSCs, derived at different ages, revealed that the main age-related alterations were found in glycolysis, fatty acid and propanoate metabolism, protein ubiquitination, valine, leucine, and isoleucine degradation pathways (Castiglione et al., 2017). For a long time, stem cells have been considered to rely only on glycolysis to fulfill their energy requirements, mainly because of a combination of their peculiar cellular demands and the hypoxic microenvironment where they reside. Nevertheless, it has become recently clear that stem cells are indeed capable of performing OXPHOS, even though glycolysis is critical for stemness maintenance, rather than being an adaptation to their environment (Maffezzini et al., 2020). *In vivo*, NPCs differentiation may be induced by the activation of a transcriptional program through NRF2, responsible for redox signaling genes expression, thereby supporting neuronal differentiation by protecting against ROS toxic insults (Lopez-Fabuel et al., 2016). Indeed, the differentiation from a pluripotent progenitor cell into a neuron is characterized by the reduced expression of glycolysis-related proteins [e.g., Hexokinase 2 (HK2) and Lactate Dehydrogenase isoform A (LDHA)], coupled to the activation of the Pyruvate Kinase PKM2, to its constitutively active isoform PKM1, and the upregulation of OXPHOS-related genes (Zheng et al., 2016). *In vitro* cultured mice SVZ-derived NSCs showed the upregulation of different energy metabolism-associated genes, such as: the Insulin-like growth factor binding protein 3 (IGFBP3), Enolase I, and Cytochrome c oxidase subunit VIIa, when compared to hematopoietic (HSCs) and embryonic stem cells (ESCs) (Ivanova et al., 2002); hypoxia-inducible factor (HIF1α), Acetyl-coenzyme A transporter, and IGFBP3, when compared to differentiated cells of the lateral ventricle (Ramalho-Santos et al., 2002; Bonnert et al., 2006). In addition, also cultured postnatal NSCs showed increased expression of metabolic genes, such as Acetyl-coenzyme A synthetase 1, Enolase I, and Pyruvate dehydrogenase, as compared to differentiating neural cells (Geschwind et al., 2001; Karsten et al., 2003), although other metabolic genes (e.g., Glucose-6-phosphate dehydrogenase) were upregulated during differentiation (Gurok et al., 2004).

**FIGURE 1:**
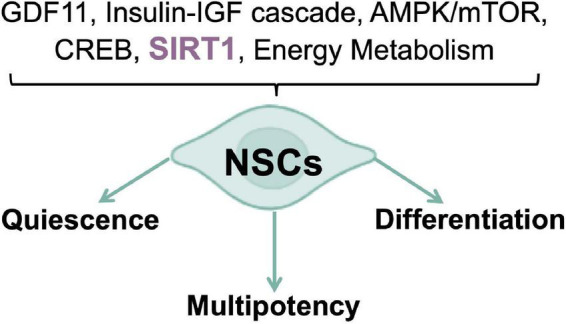
Nutrient pathways control of NSCs fate. Nutrient sensing and energy metabolism are the major physiological determinants of neurogenic processes.

Energy metabolism regulation is a crucial player in stem cell fate determination and the right balance between stem cell quiescence, multipotency, and differentiation relies on the reversible switching between aerobic and anaerobic metabolism (Beckervordersandforth, 2017). Rodents subjected to calorie restriction (CR), a regimen of calorie reduction without malnutrition, displayed an increased numbers of newly produced neural cells in the SGZ, coupled to increased expression of the brain-derived neurotrophic factor (BDNF) (Lee et al., 2000); while in models of diet-induced obesity and diabetes, adult neurogenesis results to be impaired (Pani, 2015). Furthermore, aberrant adult neurogenesis has been reported in mice models for nutrient-triggered signals impairment, thus confirming that nutrient-regulated switches influence NSCs fate decisions. The transition between quiescent and activated states is a critical step, as the required cell cycle entry is a major energetic commitment (Cavallucci et al., 2016; Wentling et al., 2019). For this reason, nutrient-responsive pathways and transducers, such as the growth differentiation factor-11 (GDF11), the insulin-IGF cascade, the AMPK/mTOR axis, and the transcription regulators CREB and SIRT1 have been included, alongside the canonical “developmental” signals (e.g., Notch and Wnt), in the molecular networks controlling NSCs self-renewal, migration, and differentiation, in response to local and systemic inputs (Figure 1). In the context of metabolic diseases as well as in aging, many of these nutrient-related cascades prove to be dysregulated, thus suggesting a possible explanation/contribution to both impaired neurogenesis and the cognition defects observed (Katsimpardi et al., 2014; Fidaleo et al., 2017). Recently, a role for short chain fatty acids (SCFAs) in adult neurogenesis has been proposed. SCFAs are the major product of fiber fermentation in the large intestine and, after entering systemic circulation, could directly impact on mitochondrial metabolism in diverse body’s tissues, including the brain (van de Wouw et al., 2018; Silva et al., 2020). Specific diet regimens could increase SCFAs levels which in turn could trigger mitochondrial biogenesis and premature differentiation of NSCs, through a ROS- and ERK1/2-dependent mechanism, thus resulting into a rapid depletion of the NSCs pool (Ribeiro et al., 2020). Therefore, nutrients are necessary to trigger neurogenesis but chronic overnutrition and/or metabolic imbalances, leading to an impaired nutrient signaling in the brain (Soto et al., 2018), could potentially result into NSCs exhaustion, thus accelerating brain aging, and altering neurobehaviors (Soto et al., 2018; Ribeiro et al., 2020). These observations suggest the existence of a new role for mitochondria, as mediators of the gut microbiota-brain axis, able to respond to dietary influences. Of note, the role of nutrients in NSCs activation is not in conflict with the established notion that CR enhances mammalian neurogenesis, as previously discussed. In fact, starvation/refeeding cycles are likely to synchronize NSCs, optimizing their refeeding response and re-entry into a quiescent state. Intriguingly, feeding cycles are in strict relationship with the circadian clock (Asher and Schibler, 2011) and a NSCs quiescence maintenance defect has been reported in mice model devoid of key molecular clock proteins (Bouchard-Cannon et al., 2013). Indeed, when normal human fibroblasts were synced *in vitro*, by means of two different protocols, rhythmic oscillations of mitochondrial respiration and glycolytic activity have been observed (Pacelli et al., 2019). Conversely, PD patients-derived fibroblasts, carrying Parkin gene mutations, showed a severe dampening of the bioenergetic oscillatory patterns, associated with a dysregulation of core clock genes expression, which was also confirmed in iPSCs and in the induced neural stem cells (iNSCs). These results highlight the existence of a reciprocal interplay between mitochondrial energy metabolism and clockwork machinery, pointing to a Parkin-dependent mechanism of regulation, and a greater level of complexity in PD pathophysiology, that could eventually be a common feature of other NDs (Pacelli et al., 2019). This evidence agrees with those emphasizing the conserved nature of diurnal variation of redox control in eukaryotes, where key cellular activities depend upon the coordination between the NAD-dependent control of metabolism and the NADPH/H_2_O_2_ control of the redox proteome spatiotemporal organization (Jones and Sies, 2015). Several evidence suggest that energy metabolism and nutrient sensing are the major physiological determinants of NSCs fate, as well as modulators of the entire neurogenic process. Nevertheless, the molecular pathways underlying metabolic regulation of neurogenesis, are still poorly understood. Their full comprehension, as well as their interplay with novel dietary and/or pharmacological approaches, aiming at improving neurogenic activity and delaying its age-related decline, may be of help in the prevention of neurodegenerative disorders and brain aging (Cavallucci et al., 2016).

### 3.2. ROS metabolism in the cell

Oxidative eustress has been defined as a “low physiological exposure to prooxidants,” characterized by the involvement of specific redox signaling targets, while oxidative distress as a “supraphysiological exposure to prooxidants,” with unspecific targets, disrupted redox signaling, and damaging molecule modifications (Lushchak and Storey, 2021; Sies, 2021). Enzymatical or non-enzymatical ROS generation could occur in different cellular compartments, such as mitochondria, peroxisome, endoplasmic reticulum (ER), and lysosome (Malard et al., 2021). Nevertheless, approximately 90% of cellular ROS are generated by the mitochondrial Electron Transport Chain (ETC) during ATP production (Nissanka and Moraes, 2018). In fact, about 0.1–0.2% of the O_2_ used by mitochondria is thought to generate ROS, through the premature electron flow deriving from NADH or FADH_2_ to O_2_, mainly through electron transport chain complexes I and III (Chen et al., 2003; Tahara et al., 2009). ROS-dependent generation of O_2_-derived reactive species is initiated by the conversion of O_2_ to O_2_^.–^ and the formation of H_2_O_2_, hydroxyl radicals OH^.^, OH^–^, and many other derivative species (Olson et al., 2017). It is very unlikely that O_2_^.–^ can play a role in physiology, especially considering its very short lifetime (approximately 10^–9^ s), although it can possibly induce oxidative damage in the neighboring area. Therefore, superoxide dismutase 1 (SOD1) is more likely to act as a signaling rather than antioxidative enzyme, because it converts O_2_^.–^ to the more stable H_2_O_2_, which can be transported as a signaling molecule. However, H_2_O_2_ is dangerous for the cell, as by the Fenton reaction it could generate hydroxyl anion, the most toxic form of ROS (Angelova and Abramov, 2018). These activation/deactivation cycles of the H_2_O_2_ metabolism support complex time-dependent processes in the life cycle of cells and organisms, as described by third principle of the redox code (Jones and Sies, 2015). In mitochondria, O_2_^.–^ generation from O_2_ depends upon the activity of different proteins, such as glycerol 3-phosphate dehydrogenase and 2-oxoglutarate dehydrogenase. Other sources of ROS are: (i) the peroxisomes, through the activity of fatty acid β-oxidation, iron-Sculpture flavin hydroxylase xanthine oxidoreductase (XOR), and urate oxidase (UO), in the matrix, and by electron chain, in the membrane; (ii) the ER, by nicotinamide adenine dinucleotide phosphate (NADPH) oxidases (NOXs) and dual oxidases, and the microsomal monooxygenase (MMO) system, which contains cytochrome P450 oxidase (Malard et al., 2021). In addition, non-home iron containing dioxygenases, lipoxygenases, and cyclooxygenases also generate O_2_^.–^ from polyunsaturated fatty acids (PUFAs) (Snezhkina et al., 2019). O_2_^.–^ reduces key transition metal ions iron Fe^3+^, copper Cu^2+^, and manganese Mn^3+^, which generate O_2_ (Hayyan et al., 2016). Under physiological conditions, ROS levels are tightly regulated by the ROS scavenging system, through the activity of antioxidant enzymes that can neutralize ROS by directly reacting with as well as accepting electrons from them. Glutathione Peroxidases (GPXs), through GSH/GSSG metabolism, and Peroxiredoxin (PRDX) generate H_2_O from H_2_O_2_ (Angelova and Abramov, 2018), with a circadian variation (Jones and Sies, 2015). Other two important antioxidant defense enzymes are Catalase and the home containing enzyme Myeloperoxidase (MPO). Catalase, the most abundant peroxisomal antioxidant enzyme, dismutates H_2_O_2_ to O_2_ and H_2_O (Ogino et al., 2001; Olson et al., 2017), while MPO catalysis the oxidation of Cl^–^ by H_2_O_2_ to hypochlorous acid (HOCl) (Garai et al., 2017). In addition, H_2_O_2_ reacts with transition metal ions Fe^2+^ or Cu^+^ to generate OH^.^, OH^–^, and the corresponding reactive Fe^3+^ or Cu^2+^ in the Fenton reaction (Kajarabille and Latunde-Dada, 2019).

Proteins belonging to the NOX family are the second major source of intracellular ROS, and this enzymatic ROS synthesis contributes toward the maintenance of physiological ROS levels, accordingly to cellular demands (Bedard and Krause, 2007). In cultured hippocampal neurons, NOX enzymes are responsible for the generation of almost 45% of intracellular hydrogen peroxide, thus proving the key role of these enzymes’ activity on the redox state of the cell (Bedard and Krause, 2007). The NOX-produced superoxide anion is rapidly converted, either spontaneously or enzymatically (by SOD), into hydrogen peroxide, which plays an important role in killing pathogens, although excess superoxide can lead to oxidative stress and cell damage. ROS accumulation leads to oxidative stress, which produces adverse effects on multiple cellular components, including proteins, lipids, and nucleotides. ROS generation in the mitochondrial matrix leads to damage of mitochondrial proteins, membranes, and DNA. Additionally, ROS can impair ATP synthesis thus impacting on metabolic functions, such as fatty acid oxidation, TCA cycle, urea cycle, amino acid, and home synthesis (Zorov et al., 2014). Moreover, oxidative damage can result in cytochrome *c* release, through the mitochondrial permeability transition pore, thus leading to apoptosis induction (Rao et al., 2014). We can thus assume that ROS levels act as a function of mitochondrial respiration and that multiple factors, such as oxygen availability, NADH, FADH2, and ubiquinone redox states, antioxidant factors activity, mitochondrial morphology, as well as mutations in OXPHOS subunits, could influence ROS levels as well (Maffezzini et al., 2020).

The brain is one of the most metabolically active among all organs, highly susceptible to stresses, specifically to oxidative distress, due to the multifaceted roles of ROS, especially superoxide anion O_2_^.–^ and H_2_O_2_, in redox signaling. Indeed, dopamine metabolism, *via* monoamine oxidases (MAO), and OXPHOS are important sources for H_2_O_2_ generation, that could behave as a signaling molecule. In addition, the brain shows low endogenous antioxidant defense, as compared to other organs, mainly due to its reduced glutathione (GSH) content and low catalase. Further, enriched unsaturated lipids, such as omega-3 docosahexaenoic acid, cause distress due to their susceptibility to lipid peroxidation related to cell death, including ferroptosis (Cobley et al., 2018). Finally, brain susceptibility is region- and time-dependent, based on the condition that induces oxidative distress and the endogenous antioxidant capacity. In fact, while many neuronal subtypes can cope with oxidative stress rise, selected neuronal populations showed a higher susceptibility to ROS increase (Mattson and Magnus, 2006; Wang and Michaelis, 2010). The four areas of the brain most susceptible to oxidative stress are the cerebral cortex, the hippocampus, the striatum, and the cerebellum (Malard et al., 2021). These observations agree also with the evidence that oxidative stress and poor antioxidant defense, underlying the deleterious effects of vitamin B12 deficiency in mice model, would act on the expression of histone modifying enzymes that act on the behavior (Ghosh et al., 2020).

Even though high ROS levels associate with cellular dysfunction, it is now evident that ROS are necessary for some physiological cellular functions (Sart et al., 2015; Nissanka and Moraes, 2018). Indeed, ROS act as second messengers by modulating cytokines and growth factors, whose activity regulates classical signaling cascades under both physiological and stress-related conditions, such as ERK, JNK, MAPK, and JAK/STAT pathways (Simon et al., 1998). Furthermore, the regulatory effect of ROS might be modulated not only by classical ROS-scavenging enzymes, such as SOD, catalase, peroxiredoxins (PRX), thioredoxin (TRX), glutathione peroxidase (GPX), reductase (GR), and transferases (GST), but also by newly identified ROS suppressors, including PTEN-induced putative kinase 1 (PINK1) and SIRT1 (Bigarella et al., 2014; Xavier et al., 2016).

## 4. Role of mitochondria in neural stem cell commitment: The SIRT-ROS interplay

**FIGURE 2:**
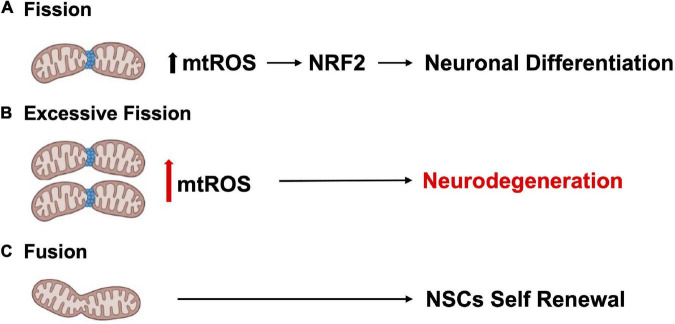
Mitochondrial dynamics regulate neurogenesis. **(A)** Mitochondrial fission promotes neuronal differentiation. **(B)** Excessive fission and ROS production contributes to neurodegeneration. **(C)** Enhanced mitochondrial fusion promotes NSCs self-renewal.

**FIGURE 3:**
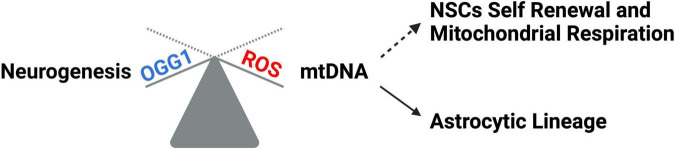
mtDNA impact on neural differentiation. ROS increase leads to mtDNA damage which correlates with NSCs self-renewal reduction, decreased mitochondrial respiration, and a shift toward the astrocytic lineage. OGG1 repair system protects NSCs from ROS-dependent mtDNA damage, thus favoring neuronal differentiation.

Tissue development and regeneration rely on the balance between stem cell self-renewal vs. differentiation, and several reports highlighted mitochondria as a key player, with functional alterations of the organelle leading to stem cell abnormalities (Llorens-Bobadilla et al., 2015; Shin et al., 2015; Stoll et al., 2015; Xie et al., 2016; Zheng et al., 2016; Khacho et al., 2019). The hGFAP-SDHD mouse model, bearing homozygous deletion of the succinate dehydrogenase subunit D gene (SDHD) restricted to the cells of glial fibrillary acidic protein lineage, showed that the genetic mitochondrial damage did not alter the generation, maintenance, or multipotency of glia-like NSCs. However, a differentiation impairment of neurons and oligodendrocytes, but not of astrocytes, has been observed alongside with extensive brain atrophy (Diaz-Castro et al., 2015). Another study proved the Nestin-Cdk5-Drp1 axis as a negative modulator of OXPHOS, which is indispensable for neural stem/progenitor cell maintenance (Wang J. et al., 2018), and it has also been reported that, during embryonic neurogenesis, NSCs’ mitochondrial morphology acts as an upstream regulatory mechanism for stem cell fate decisions (Rodolfo et al., 2016; Bueler, 2021). Indeed, enhanced mitochondrial fusion promotes NSCs self-renewal, while mitochondrial fragmentation commits NSCs to neuronal differentiation and maturation (Figures 2A, C). Khacho et al. (2016) observed a reduction of uncommitted Sox2^+^ NSCs and immature DCX^+^ neurons in the DG, when the mitochondrial fusion proteins MFN1/2 were knocked out in adult hippocampal NSCs. Indeed, when MFN2, OPA-1, and Drp1 were downregulated in Sox2^+^ NSCs, they observed aberrant mitochondrial dynamics as well as an impairment of NSCs self-renewal and fate decisions, linked with changes in ROS signaling but not in ATP levels. Mitochondrial dynamics also seem to regulate neurogenesis in the adult SVZ, as suggested by chemical inhibition (with Mdivi-1) of Drp1 in SVZ-derived neurosphere cultures, which results in a reduction of both NSCs migration form neurosphere as well as their differentiation into neurons (Detmer and Chan, 2007). This evidence showed that mitochondrial morphology changes could regulate mitochondrial metabolism and ROS generation, whereby the commitment of NSCs to a progenitor fate is mediated by a physiological increase in mitochondrial ROS (mtROS), associated with mitochondrial fragmentation (Khacho and Slack, 2018; Bueler, 2021; Ozgen et al., 2022). Mitochondrial fragmentation fulfils a biological role to regulate neuronal development (Khacho and Slack, 2018) and mtROS would act as a rheostat to direct gene expression changes regulating cell fate decisions (Maryanovich and Gross, 2013). Indeed, the physiological mtROS increase functions as a signaling mechanism to activate a cascade of events leading to the stabilization of the master redox regulator protein NRF2, whose translocation into the nucleus mediate the transcription of genes responsible for differentiation induction and self-renewal suppression (Khacho et al., 2016). On the other hand, ROS production and excessive fission are responsible for neurodegeneration and are detrimental for neurogenesis (Rodolfo et al., 2016; Bueler, 2021; Figure 2B). In this scenario, Sirtuins may act as potential modulators of specific gene activation, leading to neural differentiation. In fact, oxidative stress, or a general alteration of cellular redox homeostasis, impacts on SIRTs activity at different levels: (i) by inducing or repressing SIRTs genes expression; (ii) by posttranslational oxidative modifications of SIRTs; (iii) by altering SIRTs-protein interactions; (iv) by changing NAD^+^ levels (Santos et al., 2016). Moreover, recent evidence highlights a molecular linkage between mitochondrial DNA (mtDNA) integrity and the modulation of neural differentiation, suggesting another way by which ROS can modulate stem cells differentiation. Many pathological insults can affect mtDNA integrity, but ROS-dependent oxidative damage is the most discussed (Nissanka and Moraes, 2018). It has been reported that increased mtDNA mutation loads correlate with a reduction of NSCs population in the SVZ of adult mice, reduced self-renewal capacity, and decreased mitochondrial respiration (Ahlqvist et al., 2012; Nissanka and Moraes, 2018; Figure 3). Moreover, during brain repair, mtDNA damage was shown to favor NSCs differentiation into astrocytes, and to affect mitochondrial DNA transcription, and replication (Xavier et al., 2016). Cells developed different strategies to abolish the deleterious consequences of ROS on DNA. Indeed, a multienzymes repair cascade, known as the base excision repair (BER), leads to mtDNA repair and replication. In this cascade, OGG1 activity triggering, allow for the identification and elimination of several base lesions, including 8-oxoguanine, one of the most abundant genomic base modifications generated by reactive oxygen and nitrogen species. OGG1 acts as a transcription modulator, which can control transcription factor homing, induce allosteric transition of G-quadruplex structure, or recruit chromatin remodelers (Wang R. et al., 2018). In particular, OGG1 activation in the mitochondria results in the induction of the mitochondrial DNA polymerase, Pol γ (Xavier et al., 2016). Experiments in mice showed OGG1 as essential for the repair of mtDNA damage and NSCs viability, upon mitochondrial oxidative stress (Wang et al., 2011). In fact, differentiating neural cells from *ogg1*^–/–^ mice spontaneously accumulate mtDNA damage and shift their fate toward an astrocytic lineage. Interestingly, these events are associated with SIRT1 enzymatic activation, due to an increased NAD^+^/NADH ratio, similarly to what observed in wild type NSCs subjected to mtDNA damaging insults. Instead, antioxidant administration reversed mtDNA damage accumulation and increased neurogenesis in *ogg1*^–/–^ cells. Moreover, the expression of a mitochondrially targeted human OGG1 in *ogg1*^–/–^ NSCs results in the protection from mtDNA damage during differentiation, and increased neurogenesis (Wang et al., 2011; Figure 3). Similarly, it has been reported that AOX halted the neurogenic to gliogenic lineage shift during NSCs differentiation, by strongly reducing ROS generation and nuclear translocation of NRF2 and SIRT1 (Santos et al., 2013). This evidence would suggest that ROS-sensitive SIRTs activity could be modulated by alterations of the mitochondrial respiratory chain, in response to mtDNA damage, thus impacting on NSCs fate at different levels.

## 5. Redox signaling in neurogenesis

### 5.1. ROS modulation of adult neurogenesis

**FIGURE 4:**
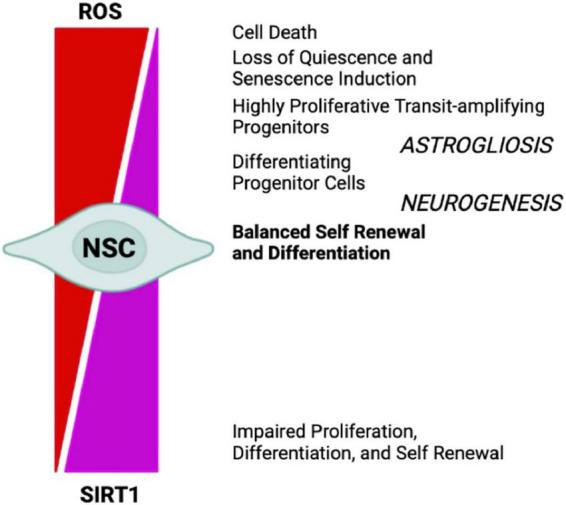
NSCs cell-fate decision depends upon redox balance and SIRTs activity. High oxidizing conditions favor NSCs differentiation into astrocytes (astrogliosis), senescence, and cell death, whereas mild oxidative/reducing conditions favor neuron formation (neurogenesis), through a complex regulatory network modulated by SIRT1 expression and activity.

Oxygen levels play a significant role in the molecular mechanisms that guide stem cells to either differentiate or renew, by directly influencing enzymatic reactions and by regulating specific gene expression profiles, through transcription factors such as HIF1α (Mootha and Chinnery, 2018). Energy generation in NSCs preferentially relies on aerobic glycolysis, through HK2 and the pentose phosphate pathway, and may be due to multiple factors, such as their location within a hypoxic niche, the low energy requirements of quiescence, and the need to minimize mtROS-dependent oxidative stress, as shown in HSCs (Kunisaki et al., 2013). The differentiation of ESCs into neural progenitors is regulated by the eicosanoid pathway and by fatty acid metabolism (Yanes et al., 2010). Indeed, stem cells’ mitochondria are relatively metabolically inactive, in terms of ATP production, when compared with more differentiated cells. Nonetheless, functional mitochondria are still required for proper adult stem cells maintenance (Bigarella et al., 2014). Stem cells maintain low basal levels of ROS, which preserves stem cell potential by maintaining an appropriate balance between stem cell quiescence, differentiation, and self-renewal. The oxidative stress response mediated by Forkhead box O 3 (FOXO3) becomes rapidly deactivated upon NSCs differentiation, suggesting that mitochondrial oxidation-induced ROS are required in neural progenitors (Shyh-Chang et al., 2013). In fact, in NSCs and HSCs ROS reduction below the basal level is associated with reduced regenerative potential, characterized by impaired proliferation, differentiation, and self-renewal (Collins et al., 2018; Tan and Suda, 2018). However, high levels of H_2_O_2_ are required to maintain regular NSCs and progenitor cells’ self-renewal, a process related to Doublecortin (DCX), Nestin, and FOXO proteins (Le Belle et al., 2011). Conversely, ROS accumulation leads to loss of quiescence and induction of senescence, *via* p38-MAPK activation, leading to stem cell exhaustion and impaired regenerative potential, which could be reverted by administration of the antioxidant N-acetylcysteine (NAC) (Ito et al., 2006; Paik et al., 2009; Takubo et al., 2010; Ahlqvist et al., 2012; Borodkina et al., 2014; Garcia-Prat et al., 2016; Shaban et al., 2017; Tan and Suda, 2018). Further, accumulation of ROS to high levels ultimately results in cell death (Sart et al., 2015; Tan and Suda, 2018). On the other hand, it has been reported that p53-dependent mtROS reduction impacts on the neural differentiation potential, by favoring neuronal rather than astroglial conversion (Xavier et al., 2014). In the SGZ, transient oxidative distress, stimulates the expression of oxidation-responsive genes, which in turn drive neurogenesis by promoting NSCs and progenitor cells differentiation (Walton et al., 2012). However, a recent study in which mice NPCs from hippocampus were FACS-sorted accordingly to their ROS levels, and subjected to staminal markers and transcriptome analyses, unexpectedly showed that the cells with the highest ROS levels were quiescent NSCs (qNSCs). Shifts to lower ROS content primed NPCs to a subsequent state transition, showing that lower ROS levels correlated with increased expression of proliferation and differentiation genes. In addition, NOX2 was not necessary for NPCs proliferation under physiological conditions, even if it has been reported that a transient NOX2-dependent ROS burst promotes exercise-induced recruitment of qNSCs to proliferation (Adusumilli et al., 2021). While this evidence seems to be in contrast with previous ones, they may not be mutually exclusive, because the transient nature of ROS and ROS signals likely triggers cell transitions without substantially altering ROS levels in the next cell type, especially if the ROS burst also activates anti-oxidative genes (Bueler, 2021). Interestingly, from the antioxidant defense side, *in vivo* experiments in mice deficient for cytoplasmic (SOD1) and mitochondrial SOD (SOD2), showed reduced adult hippocampal neurogenesis, in favor of an increased generation of new-born astrocytes (Garcia-Prat et al., 2016). In addition, NSCs and NPCs proliferation were increased by the accumulation of catalase in mitochondria (Liao et al., 2013). Finally, MPO inhibition increases adult neurogenesis through, at least, DCX and SOX2 stimulation (Yu et al., 2018). ROS behavior during neurogenesis, is like that described in the Hekimi’s Mitohormesis theory (Hekimi et al., 2011), which states that cellular insults trigger protective stress response, where ROS would act as secondary messenger. Several key transcription factors and signaling pathways, including NOTCH and WNT/β-catenin, NRF2, p53, PI3K/AKT, and pERK1/2 (Bouchard-Cannon et al., 2013), are involved in the ROS-dependent modulation of both adult and embryonic neurogenesis. Therefore, ROS can act as signaling molecules to modulate the stress response pathway and small increases can extend lifespan. However, at a certain point this age-dependent damage would increase past the threshold where ROS signaling is sustained and maladaptive (Figure 4).

### 5.2. SIRT-mediated ROS-dependent neurogenesis

**FIGURE 5:**
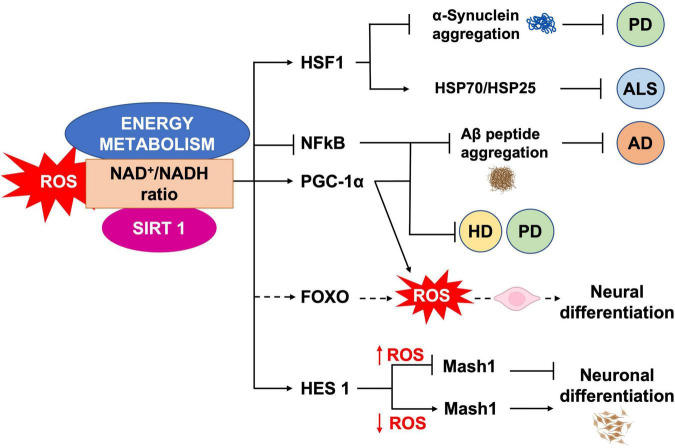
ROS, SIRT1 and energy metabolism interplay. NAD^+^/NADH ratio is a measure of the cellular redox status, and plays a role in NSCs fate regulation, through SIRT1 activity. SIRT1 regulates neural differentiation as well as cellular pathways related to NDs, by regulating the activity of different transcription factors.

It has been shown that mild oxidative stress triggers SIRT1 activation and subsequent HES-1 mediated transcriptional inhibition of MASH1, leading to increased astrogliogenesis (Prozorovski et al., 2008). Additionally, mouse models of advanced aging, bearing a deletion in the clock gene *BMAL1*, show high levels of ROS and SIRT1 expression in the brain, coupled with a reduction of hippocampal adult neurogenesis in favor of an increased production of astrocytes (Ali et al., 2015). SIRT1 activation is crucial for mitochondrial homeostasis, as it regulates the expression of OXPHOS enzymes as well as of the peroxisome proliferator-activated receptor γ (PPARγ) coactivator-1a (PGC-1α), which is crucial for mitochondrial gene expression (Lin J. et al., 2005). NAD^+^/NADH ratio, which is in turn a measure of cellular redox status (Jones and Sies, 2015), plays a role in regulating stem cell fate through SIRTs activity (Haigis and Sinclair, 2010; Imai and Guarente, 2014, 2016). During aging, the oxidative stress increase determines a NAD^+^ depletion that negatively impacts on mitochondria (Du et al., 2003). *In vivo* mice model of chronic cerebral hypoperfusion, showed that NAD^+^ improved cognitive function and reduced neuroinflammation in association to mitochondrial protection and ROS inhibition through the activation of SIRT1/PGC-1α pathway (Zhao et al., 2021). Moreover, NAD^+^ levels depend upon the energetic level of the cells, increasing during CR and decreasing under conditions of high-energy load, such as high-fat diet. Interestingly, CR decreases oxidative stress leading to increased NAD^+^ levels and improving mitochondrial function, by the SIRT3-mediated increase of SOD2 activity (Qiu et al., 2010). Therefore, NAD plays a role in the mito-nuclear protein imbalance, which has been described as a conserved mechanism in the regulation of energy metabolism (Houtkooper et al., 2013). Thus, as SIRTs enzymatic activity depends upon NAD^+^ levels they could act as metabolic sensors coupling cellular metabolic status to regulatory responses (Nemoto et al., 2004; Canto and Auwerx, 2009; Peng et al., 2010; Khoury et al., 2018). It has been shown that ROS levels also influence the oxidation state of cysteine (Cys)-containing redox sensors, following a diurnal variation (Blanco et al., 2007), which alter the activity and localization of these proteins, thereby regulating NSCs state and fate. SIRT1 contains critical cysteine residues vulnerable to oxidation, whose alteration decreases enzyme’s activity and favors its degradation (Cai et al., 2012; Chen et al., 2012). Summarizing, pathological ROS balance alteration as well as their age-associated physiological accumulation, may affect SIRTs expression and/or activity and in turn NSCs fate decisions. It would be of outmost importance to better clarify the exact molecular mechanisms linking SIRTs enzymatic activity and ROS-dependent modulation of the correct lineage specification and/or aging in NSCs (Figure 5).

## 6. SIRT proteins in neurogenesis and CNS aging-related diseases

**FIGURE 6:**
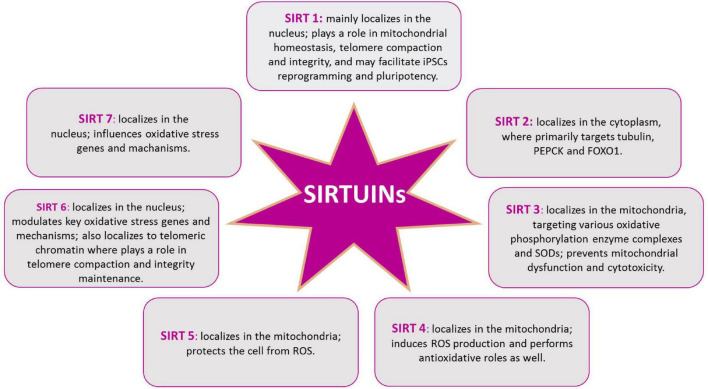
SIRTs protein family. Intracellular distribution of SIRTs in the cytoplasm, the mitochondria, and the nucleus, allows them to play different roles in cellular redox homeostasis.

In mammals, beside the above described SIRT1, seven SIRTs (SIRT1-7) have been identified, all possessing a highly conserved central nicotinamide adenine dinucleotide (NAD^+^)-binding site and a conserved catalytic domain. SIRT1 mainly localizes in the nucleus; SIRT2 in the cytoplasm, where primarily targets tubulin, PEPCK and FOXO1; SIRT3, SIRT4, and SIRT5 in the mitochondria, targeting various oxidative phosphorylation enzyme complexes and SODs; SIRT6 and SIRT7 in the nucleus, with SIRT6 targeting histone H3, PARP-1, and p65 (Walton et al., 2012; Maissan et al., 2021; Figure 6). Recently, SIRTs have been reported to also modulate neurodegeneration and toxicity associated with different proteins such as α-synuclein (α-SYN), huntingtin (HTT), TAU, or amyloid-beta (Aβ) peptide (Donmez, 2012). The possibility to direct NPCs differentiation may be useful to protect brain against inflammatory diseases, such as MS, which involves astrogliosis. In addition, the ability to precisely direct NPCs differentiation toward neurons may provide new therapeutic options for stroke, spinal cord injury, and age-related cognitive conditions, characterized by neuronal loss, such as AD and PD (Watroba et al., 2017). Aging is the major risk factor for NDs development and aging stem cells lose their ability to produce NPCs as well as their differentiation capacity, as described above. The conspicuous presence of SIRTs in the brain and the importance of their role in mitochondrial metabolism regulation, suggest this proteins family as a good target candidate for therapeutic protocols, aiming to control neurogenesis and NDs (Figure 5). SIRTs-dependent modulation of pluripotency and differentiation is not entirely understood, but it has been shown that SIRT1, 2, 3, 6, and 7 were involved in the modulation of these processes (Karuppagounder et al., 2015; Correia et al., 2017). In this review, we mainly focused on SIRT1 role in CNS, as this is the most studied member of the family (Mishra et al., 2021). *In vitro* and *in vivo* observations showed SIRT1 as required for proper differentiation of both ESCs and adult stem cells. Indeed, SIRT1 expression, which is higher in ESCs, decreases during differentiation, through a miRNA-mediated post-transcriptional regulation (Saunders et al., 2010; Lee et al., 2019). In addition, oxidative stress and inflammation can promote NPCs differentiation toward the astrocyte lineage, through a SIRT1-dependent regulation of the *MASH1* promoter1. It has been shown that the redox state does affect NPCs cell-fate decision *in vitro*, with oxidizing conditions favoring differentiation into astrocytes, whereas reducing conditions favor neuron formation (Libert et al., 2008). In fact, administration of the SIRT1-activating compound resveratrol to NPCs mimicked oxidizing conditions and increased NPCs differentiation toward astrocytes, through a SIRT1-dependent mechanism (Figure 4). Under oxidizing conditions, SIRT1 and the hairy and enhancer of split 1 (HES1) form a complex that binds to and deacetylates histones at the *MASH1* promoter, while recruiting co-repressors, such as TLE1 (Libert et al., 2008). Together, these events cause a down-regulation of MASH1 expression and block neuronal differentiation. The HES1-SIRT1 complex is not detected under reducing conditions, where HES1 could recruit transcription activators, such as CREB binding protein (CBP), to the *MASH1* promoter, driving NPCs differentiation toward a neuronal fate (Prozorovski et al., 2008). In a recent work, it has been reported that extracellular glucose, through the coordinated action of CREB and SIRT1, could modulate HES1 expression in NSCs and NPCs. Indeed, excess glucose reduced CREB-activated HES1 expression and resulted in impaired cell proliferation. Moreover, CREB-deficient NSCs expanded poorly *in vitro* and did not respond to glucose availability. Elevated glucose levels also promoted SIRT1-dependent repression of the *HES1* promoter. Conversely, in low glucose, CREB replaced SIRT1 on the chromatin associated with the *HES1* promoter, enhancing HES1 expression and cell proliferation. Thus, the glucose-regulated antagonism between CREB and SIRT1 for *HES1* transcription participates in the metabolic regulation of neurogenesis (Fusco et al., 2016). These works suggested that NSCs proliferative potential is subject to tight intrinsic regulation; therefore, a better knowledge of SIRTs role in the modulation of intracellular pathways controlling cell cycling would be of importance for the reactivation of latent NSCs populations, to engage endogenous neurogenesis, as a treatment for different NDs. *In vivo* and *in vitro* AD models showed that SIRT1 could exert a protective action toward neuronal damage (Julien et al., 2009). Indeed, SIRT1 has been shown to directly deacetylate TAU protein, thus increasing its susceptibility to degradation and preventing neurofibrillary tangles formation (Min et al., 2010; Xu et al., 2018). Moreover, autophagy-dependent Aβ degradation may also be related to SIRT1 activity (Park et al., 2016), as SIRT1 activation/overexpression has been reported to interfere with microglia-mediated Aβ toxicity, through its ability to inhibit NF-κB signaling (Chen et al., 2005). SIRT1 can also deacetylate PGC-1α, thus increasing its transcriptional regulation activity. In fact, deacetylated PGC-1α can instill transcriptional repression of β-secretase, which in turn can reduce Aβ production levels and senile plaque accumulation (Xu et al., 2018). These results are corroborated by a systematic review where it has been shown the protective role of resveratrol in AD patients (Buglio et al., 2022). Recently, the analyses of PD animal and cellular models, showed that SIRT1 overexpression was able to suppress α-SYN aggregates formation, through the activation of the molecular chaperones, driven by Heat Shock Factor 1 (HSF1) deacetylation (Donmez et al., 2012). Resveratrol was shown to have a protective effect against α-SYN-induced toxicity in SK-N-BE cells (Albani et al., 2009), and another study showed that resveratrol administration results into increased PGC-1α transcription and improved mitochondrial function, through the AMPK-SIRT1-PGC-1α signaling pathway (Ferretta et al., 2014). In PD mouse models, treatment with resveratrol and the polyphenol Epigallocatechin gallate (ECGC), results in the protection against toxicity through an up-regulation of PGC-1α, *via* SIRT1 activity (Xu et al., 2018). In addition, CR or 2-deoxy-D-glucose (2-DG) administration were found to reduce dopaminergic neurons loss in mice as well as to improve motor function (Duan et al., 2003), corroborating the involvement of SIRT1 in the longevity-modulating role of the insulin/IGF signaling (IIS) under CR (Holzenberger et al., 2003; Suh et al., 2008; Deelen et al., 2013). SIRT1 has a neuroprotective role also in HD (Duan, 2013; Smith et al., 2014). In fact, it has been observed that mutant HTT reduces SIRT1 activity, impairing its positive role in neuronal survival, probably due to the structural similarity between mutated HTT and SIRTs-interacting transcription factors (Naia and Rego, 2015). A recent study showed that, in mouse, SIRT1 improved survival, neuropathology, and the expression of brain-derived neurotrophic factor (BDNF), which requires the presence of CREB-regulated transcription coactivator 1 (Jeong et al., 2011). In HD knock in mice model, PGC-1α is repressed by mutant HTT and PGC-1α knockout exacerbates neurodegeneration and motor abnormalities. Conversely, PGC-1α expression ameliorates mitochondrial dysfunction and rescued neuronal toxicity induced by mutant HTT. In this context, SIRT1 ablation exacerbates neurodegeneration, whereas SIRT1 overexpression improves motor functions and rescued brain atrophy. As already described, PGC-1α is a transcriptional coactivator regulating several key mitochondrial processes, among which mitochondrial biogenesis and oxidative phosphorylation. Accordingly, SIRT1 protection against HD-related neurodegeneration is, at least partially, related to prevention of mitochondrial function impairment, through PGC-1α activation (Rodgers et al., 2005; Min et al., 2013). Several studies, using resveratrol administration, indicated that SIRT1 may be protective also in tissue culture and mouse models of ALS, by promoting neuronal survival. Indeed, increased SIRT1 expression levels have been reported for different brain regions in SOD1G93A transgenic mice, suggesting a role for SIRT1 in the motor functions in ALS, although the mechanisms and functional implications of this increased SIRT1 expression still require elucidation (Lee J. et al., 2012). One proposed mechanism is that SIRT1-dependent deacetylation of HSF1, results in an increased expression of molecular chaperones, like HSP70 and HSP25, that help to maintain intracellular protein homeostasis, thus reducing motor neurons toxicity (Watanabe et al., 2014); in another mechanism, SIRT1 activation results in an increased mitochondrial biogenesis, through PGC-1α and MFN2 (Min et al., 2010; Imai and Guarente, 2014, 2016; Park et al., 2016). Other works revealed that, in the ventral spinal cord, resveratrol protective effects were associated with increased expression and activation of SIRT1 and AMPK, resulting in the normalization of the autophagic flux and, more importantly, in an increased mitochondrial biogenesis (Mancuso et al., 2014). Finally, in the spinal cord of wild type mice, SIRT1 expression decreases during aging. Mouse models, either overexpressing or lacking SIRT1 in motor neurons, showed that SIRT1 slows age-related degeneration of motor neurons’ presynaptic sites at neuromuscular junctions (NMJs) (Herskovits et al., 2018).

SIRT2 is abnormally overexpressed in AD, and it is responsible for tubulin deacetylation, leading to microtubule destabilization, TAU dissociation from microtubules, and its subsequent oligomerization and aggregate formation (Silva et al., 2017). In two mouse models, SIRT2 small molecule inhibitors have been shown to reduce Aβ load and led to cognitive improvement (Biella et al., 2016). In PD, SIRT2 inhibition reduces α-SYN aggregation and toxicity, by modifying its acetylation levels (de Oliveira et al., 2017). Instead, the potential role of SIRT2 in aging is suggested by the association found between human longevity and a polymorphism in the probably regulatory elements of the SIRT2 gene (Crocco et al., 2016).

SIRT3 reacts to nutritional status and mediates some of the beneficial effect of CR, including many of the CR-induced transcriptional changes, as observed in the suppression of cochlear neurons degeneration (Someya et al., 2010). SIRT3 is upregulated when ROS are pharmacologically augmented in neuronal culture and in human AD brains, while is reduced in cells expressing mutant HTT (Weir et al., 2012). On the other hand, small molecules mediated SIRT3 upregulation decreases ROS levels and prevents mitochondrial dysfunction and cytotoxicity induced by mutant HTT (Fu et al., 2012). It has been also suggested that SIRT3 plays a role in ALS, as its overexpression protects against SOD1G93A-induced mitochondrial fragmentation and neuronal cell death (Song et al., 2013), in agreement with other works showing SIRT3 protection against aging-linked apoptosis in mice and excitotoxic insults in cultured neurons (Lee J. et al., 2012), although the exact mechanism remains elusive. It has been proposed that Cyclophilin D (CYPD), a component of the mitochondrial permeability transition pore, could be involved. Indeed, CYPD reduction delays motor neuron cell death and extends the lifespan of SOD1G93A mice (Martin et al., 2009). Notably, CYPD is a SIRT3 substrate, and SIRT3-dependent deacetylation inhibits CYPD function, prevents mitochondrial permeability transition and age-related cardiac hypertrophy (Hafner et al., 2010). In the same work, it has been shown that PGC-1α, similarly to SIRT3, is able to restore mitochondrial dynamics and cell viability of mutant SOD1G93A neurons (Song et al., 2013) and PGC-1α promotes SIRT3 expression (Kong et al., 2010). SIRT3 was also shown to physically interact with the long chain acyl-CoA dehydrogenase (LCAD) in NSCs and to require its activation to prevent age-impaired neurogenesis in mice (Santos et al., 2021). The repertoire of SIRT3 interacting partners suggest further aspects of its role also in longevity. In fact, SIRT3 deacetylation also supports the stability and activity of OGG1, thus protecting mtDNA from the accumulation of the mutagenic damage produced by 8-oxoguanine (Cheng et al., 2013); deacetylates the DNA repair regulator protein Ku70 (Sundaresan et al., 2008); binds the heat shock protein HSP70, resulting in an increased nuclear presence (Law et al., 2009). Summarizing, these interactions are potentially linked to the mechanisms of age-related neurodegeneration (Jesko et al., 2017).

Alzheimer’s disease patients showed decreased expression of SIRT6 and mice lacking SIRT6 showed TAU protein stabilization and increased phosphorylation, *via* the activation of the glycogen synthase kinase 3 (GSK3) (Kaluski et al., 2017). Like SIRT1 and SIRT3, also SIRT6 plays a role in CR (Kanfi et al., 2008). In cells under H_2_O_2_-induced oxidative stress, the suppression of SIRT6 protein levels mediates premature senescence-like phenotype (Liu et al., 2014). In turn, SIRT6 levels restoration rescues several senescence linked traits, through the modulation of IIS-mTOR signaling and restores the DNA base excision repair efficiency in human foreskin fibroblasts from aged donors (Takasaka et al., 2014; Xu et al., 2015). The links among SIRT6, DNA repair, and aging also extend to telomere maintenance. Indeed, SIRT6 localizes to telomeric chromatin where it facilitates the binding of Werner Syndrome (WS) protein, a DNA helicase crucial for genome stability. Accordingly, SIRT6 deficiency leads to replicative senescence and telomere dysfunction, resembling the WS pathology (Michishita et al., 2008). Moreover, to mitigate aging and oxidative stress SIRT6 interacts with several crucial pathways of transcriptional regulation as NRF-2 and NF-κB (Kawahara et al., 2009; Pan et al., 2016).

**FIGURE 7:**
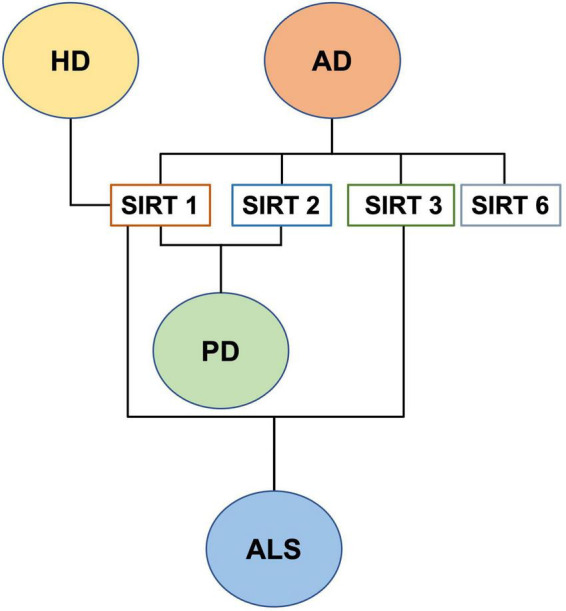
SIRTs and neurodegenerative diseases.

Lastly, insufficient data are available on mitochondrial SIRT4, SIRT5, and SIRT7 dysregulation in NDs and aging. In conclusion, although until now relatively little is known about the role of all SIRTs in neurogenesis and age-associated neurodegenerative diseases, it is becoming clear that this protein family plays a role in adult neurogenesis, controlled by mitochondrial metabolism and ROS, as well as in the antioxidative defense in the aging brain and in the aging-related CNS diseases (Singh et al., 2018; Figure 7).

## 7. Telomere shortening in NSCs and CNS aging-related diseases: SIRTs and ROS involvement and connection

**FIGURE 8:**
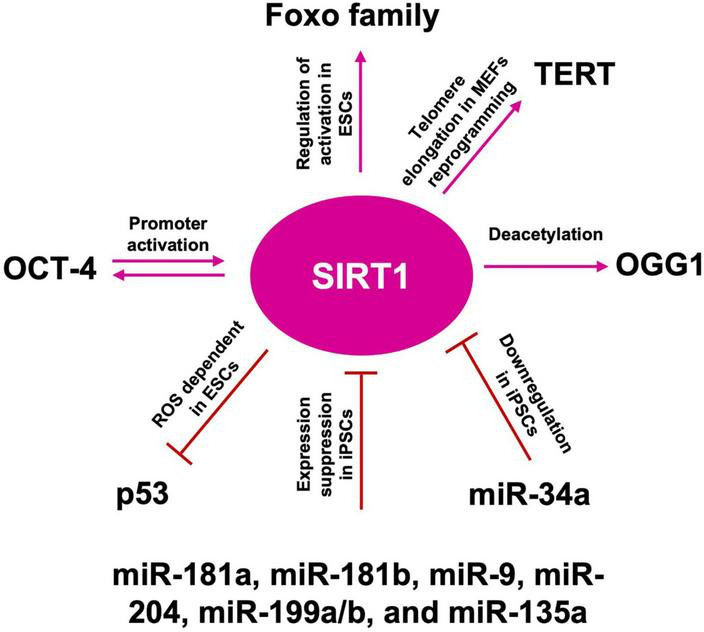
SIRT1 role in telomere length maintenance and cellular reprogramming. SIRT1: acts on OGG1 deacetylation, preserving telomere repairing; activates TERT transcription promoting telomere elongation; acts by blocking p53 nuclear translocation through ROS favoring the reprogramming; mutually interacts with OCT-4 in the cellular reprogramming process; activates FOXO family of transcription factors, which are required to maintain pluripotency; miR-181a, miR-181b, miR-9, miR-204, miR-199a/b, and miR-135a suppress SIRT1 expression, facilitating iPSCs reprogramming and pluripotency; downregulated by miR-34a promotes iPSCs differentiation into NSCs.

Telomeres are chromosome termini structures consisting of tandem DNA nucleotide repeats and the shelterin complex, a six-protein complex comprising TRF1, TRF2, POT1, TIN2, TPP1, and RAP1 (de Lange, 2018). It has been reported that in NSCs, isolated from the subventricular zone (SVZ) of telomerase-deficient adult mice, telomere attrition dramatically impairs *in vitro* proliferation. In addition, NSCs with short telomere, showed upregulation of p53 expression, in agreement with the importance of p53 pathways in the telomere damage response in mice (Ferron et al., 2004). SIRT1 overexpressing mice showed increased health span and longer telomeres, as compared to both wt and SIRT1-deficient mice, the latter showing even shorter telomeres. SIRT1 overexpression prevents telomere shortening, as the mice grew older, through the stimulation of the telomerase enzyme, whose activity is the major contributor to telomere production (Palacios et al., 2010). Moreover, SIRT1 may also influence a second telomeres maintenance pathway, called alternative lengthening of telomeres (ALT). In fact, SIRT1 overexpression increased the amount of homologous recombination, a key step in the ALT pathway, all along the chromosomes and at chromosome ends. Accordingly, SIRT1-deficient cells showed increased damage at their chromosome ends (Palacios et al., 2010) and SIRT1-overexpressing mice stay healthier for longer (Short, 2010). Notably, it has been also shown that SIRT1 binds to the elongated telomeres of differentiated cells reprogrammed into an embryonic stem cell-like state (Palacios et al., 2010). Resveratrol and progesterone can mediate telomerase activity in self-renewing human cells, with resveratrol activating, and progesterone inactivating the enzyme. Nevertheless, a direct connection between telomerase activity modulation and SIRT1 activity has not been shown yet (Pearce et al., 2008; Koziel et al., 2011). A study reported longer leukocyte telomere length (LTL) in PD patients (Pearce et al., 2008) while others, examining LTL in psychological stress, cognitive impairment, and dementia, found shorter LTL associated with these conditions (Eitan et al., 2014). In a recent study, whole genome sequencing of ALS patient’s leukocyte-derived DNA, revealed longer telomeres, in agreement with observations in PD patients (Al Khleifat et al., 2019). Another work showed a trend for longer telomeres in microglia from human post-mortem brain tissue with ALS (Linkus et al., 2016). However, the same authors found that knocking out telomerase in *SOD1G93A*-transgenic mice accelerated the ALS phenotype, concluding that telomerase dysfunction might contribute to the age-related risk for this disease. As discussed above, during normal aging as well as in NDs an increase of oxidative stress in neurons and glial cells is observed, but it is still unknown neither if oxidative stress causes telomere erosion, nor if ROS-induced telomere shortening in neurons and glia is a causal or contributing factor to NDs. As reviewed by Eitan et al. (2014), short telomeres in immune cells, astrocytes, and neurons could enhance oxidative stress-dependent senescence as well as the associated secretion of pro-inflammatory mediators (senescence-associated secretory phenotype), that may enhance disease progression. Moreover, *in vitro* cultured neurons showed that telomere damage can trigger cell death (Jurk et al., 2012; Stephenson et al., 2018) while telomerase activation may reduce neuronal vulnerability (Cheng et al., 2007; Eitan et al., 2012). In aged primary cells, increased ROS, caused by progressive mitochondrial failure, is concomitant with telomere shortening (Sanderson and Simon, 2017) and ROS neutralization does not restore mitochondrial function but still inhibits telomere shortening, thus suggesting ROS as the main player in telomere shortening (Billard and Poncet, 2019). One possible explanation for this ROS-dependent event relies on the presence of telomeric GGG repeats. ROS influence the GGG repeats by generating stretches of 8-oxoguanine, especially difficult to repair. The presence of unrepaired single or tandem 8-oxoguanine drastically inhibits TRF1 and TRF2 binding, thus impairing telomerase recruitment and contributing to telomere deprotection and shortening. Indeed, when oxidative stress is combined with telomerase inhibitors, it results into faster telomere shortening, only in oxidative damage repair deficient cells (von Zglinicki, 2002; Billard and Poncet, 2019). To counteract the deleterious telomeric consequences of ROS production, cells exploit OGG1 activity, which plays an important role also at telomeres, beside the mitochondrial one already described above. In fact, it has been reported that OGG1 depletion results into chronic replication stress and an increased telomere loss (Billard and Poncet, 2019). As OGG1 activity modulation depends upon SIRT1-dependent deacetylation, it is reasonable to hypothesize that SIRT1 plays a role in the repair of telomeric 8-oxoG in hippocampal cells (Sarga et al., 2013; Figure 8). SIRT1 also plays a role in telomere compaction and integrity, as showed by the attenuation of telomere shortening during aging, in SIRT1 gain of function mouse model, and by the reduction of H3K9me3 and fragile telomeres, in SIRT1 depletion model (Palacios et al., 2010). Finally, SIRT6 is responsible for H3K9 deacetylation at telomeres, and its depletion leads to replication defects, unrepaired DNA damage, and an accelerated aging phenotype. Moreover, SIRT6 facilitate the binding of the DNA helicase WRN, crucial for genome stability, to telomeres while SIRT6 deficiency leads to replicative senescence and telomere dysfunction, resembling the pathology seen in WS (Michishita et al., 2008). To mitigate aging and oxidative stress, SIRT6 interacts with several crucial pathways of transcriptional regulation, such as NRF-2 and NF-κB (Kawahara et al., 2009; Pan et al., 2016). All these data confirm the interplay between ROS levels and SIRTs also in the modulation and protection of telomere length, a key factor for NSCs self-renewing, protection from NDs onset, and brain aging.

## 8. SIRTs in cellular reprogramming

### 8.1. SIRT1-mediated telomere elongation

Somatic cells reprogramming, by the forced expression of Yamanaka factors (OCT-4, KL-4, SOX-2, and c-MYC), enables the generation of iPSCs, displaying ESCs-like properties (Takahashi et al., 2007). After reprogramming, also somatic mitochondria can revert to an ESC-like state in terms of morphology, cellular distribution, and rate of biogenesis (Prigione et al., 2010). Both iPSCs and ESCs are characterized by a low redox status and by the capability to repair their DNA, following oxidative damage (Armstrong et al., 2010). However, the iPSCs reprogramming protocol, through viral transduction, is associated with high ROS generation, leading to oxidative damage, impaired cell survival, and increased genetic aberrations. Administration of AOX, such as NAC or vitamin C, improves reprogramming efficiency and reduces genetic abnormalities (Ji et al., 2014), thus validating the paradigm that also during the cell reprogramming, balanced levels of oxidative phosphorylation must be maintained on the route to pluripotency (Skvortsova et al., 2022). Telomere elongation is an iPSCs hallmark, thus the role of SIRTs in pluripotency maintenance has been extensively investigated. As described above, in iPSCs SIRT1 is recruited to the telomeres and binds the telomeric repeats (Palacios et al., 2010). In murine embryonic fibroblasts (MEFs) reprogramming, SIRT1 is required for efficient post-reprogramming telomere elongation, through a c-MYC-dependent regulation of the mTERT gene, and SIRT1-deficient iPSCs accumulate chromosomal aberrations and show a de-repression of telomeric heterochromatin (De Bonis et al., 2014). Moreover, it has been shown that SIRT1 plays a role in the maintenance of iPSCs also after the acquisition of pluripotency (Zhang et al., 2014). In late passages iPSCs, SIRT1 slows down c-MYC degradation, thus ensuring enough binding to the TERT promoter and increasing TERT transcription and expression (Wu et al., 1999; Figure 8).

### 8.2. SIRT1-p53 regulatory axis

It has been reported that reprogramming, by classic Yamanaka factors, of human dermal fibroblasts (HDFs) from older human subjects, was more difficult than those of youngers, but that could be improved by SIRT6 expression. As of today, little is known about the molecular mechanism of SIRT6 regulation (Sharma et al., 2013). Several studies showed p53 as a negative regulator of reprogramming and that reprogramming efficiency could be ameliorated by p53 pathway’s inhibition (Gong et al., 2016; Ong and Ramasamy, 2018). In wild type mouse ESCs (mESCs), following DNA damage, p53 binding to Nanog promoter inhibits expression and results in pluripotency loss and differentiation (Lin T. et al., 2005). In wt mESCs endogenous ROS could trigger apoptosis, through mitochondrial translocation of p53 and BAX, while in SIRT1^–/–^ mESCs they promote nuclear p53 translocation and Nanog inhibition. Hence, ROS-dependent SIRT1 activation, acts by blocking p53 nuclear translocation (Han et al., 2008), thus modulating gene expression under ROS control, confirming the role of ROS as signaling molecules (Figure 8). Jang et al. (2017) showed that SIRT1 depletion in human ESCs (hESCs) results in p53 hyperacetylation and a dramatic reduction of DNA repair proteins, thus favoring DNA damage accumulation. Nevertheless, SIRT1 role as well as its interplay with different transcription factors are still under debate. In fact, even if it has been reported that OCT-4 could directly interact with and activate the SIRT1 promoter, thus in turn inactivating p53 through SIRT1-dependent deacetylation (Zhang et al., 2014), other studies showed that SIRT1 and OCT-4, along with SOX2, co-occupy the same distal enhancer region at the OCT-4 promoter, and cells lacking SIRT1 showed hyper-acetylation of OCT-4 (Williams et al., 2016). There are also controversial reports about a lack of binding between SIRT1 and the OCT-4 promoter (Chen et al., 2014), and other suggesting that SOX2 and SIRT1 interaction requires OCT-4 (Mu et al., 2015). Nevertheless, a key role for SIRT1 is suggested by the observation that SIRT1^–/–^ MEFs exhibited decreased iPSCs reprogramming efficiency, a defect that could be rescued by SIRT1 overexpression. In hESCs, SIRT1 also regulates the activation of the FOXO family of transcription factors, which are required to maintain pluripotency, by directly regulating the expression levels of OCT-4, Nanog, and SOX-2 (Zhang et al., 2011). In addition, SIRT1 downregulation has been observed during mouse iPSCs differentiation into NSCs (Hu et al., 2014). Furthermore, miRNA such as miR-181a, miR-181b, miR-9, miR-204, miR-199a/b, and miR-135a have been shown to suppress SIRT1 expression, suggesting a new strategy in the regulation of somatic cells reprogramming toward iPSCs (Hsu et al., 2018). Indeed, SIRT1 may facilitate iPSCs reprogramming and pluripotency, through the miR-34a-SIRT1-p53 axis, as SIRT1 downregulation by miR-34a, results in the inhibition of MEFs-derived iPSC formation, suggesting a possible involvement in iPSCs differentiation into NSCs. These results indicate that the early stage SIRT1 repression may contribute to the initiation of NSCs/NPCs differentiation from ESCs and iPSCs and explain, at least partially, the developmental defects observed in the CNS of SIRT1 deficient mice (Lee Y. et al., 2012; Figure 8). Despite these data highlight that the SIRT1-p53 regulatory axis plays a role also in cellular reprogramming, the real extent of SIRTs involvement in cell reprogramming needs to be further investigated. Moreover, further understanding of SIRTs involvement in age-related mitochondrial alteration, like ROS levels increase, could also help to modulate, or ameliorate stem cell reprogramming, through the usage of SIRTs modulators, such as resveratrol, or NAD^+^ modulators, as bioarginine.

## 9. Discussion

**FIGURE 9:**
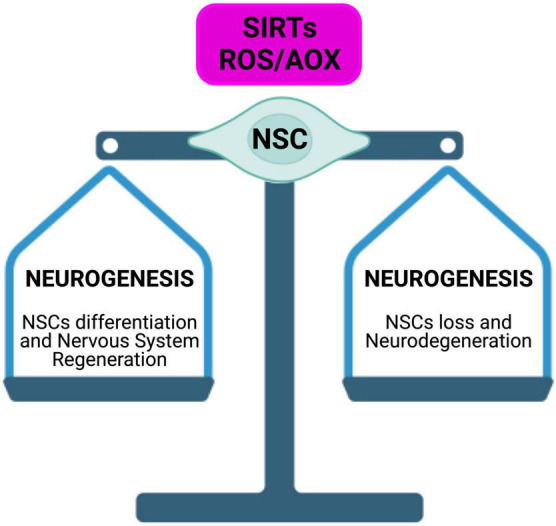
Energy metabolism regulation in NSCs fate determination. Imbalance of redox cellular homeostasis, can affect SIRTs expression levels and activity, leading to a modulation of the balance between stem cell quiescence, self-renewal, and differentiation.

**FIGURE 10:**
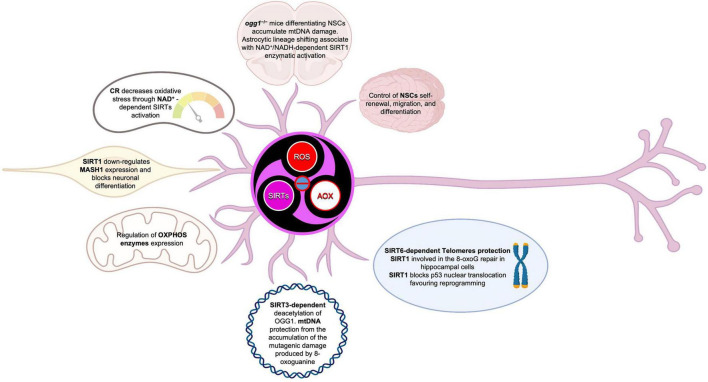
Sirtuins in the brain build up a connection between epigenetic and metabolism. SIRTs act as modulators in a complex molecular network, under direct and indirect ROS control. SIRTs-ROS/AOX balance affects NSCs fate, reprogramming, and aging, mainly through mitochondria physiology regulation and telomere protection.

The scientific evidence we reviewed here, suggested that, in the CNS, Sirtuins build up a connection between epigenetic and metabolism, by acting as modulators in a complex molecular network, under the direct and indirect ROS control, to determine NSCs fate, reprogramming, and aging, through mitochondria regulation and telomere protection. Indeed, oxidative stress, or a general disruption of redox cellular homeostasis, can affect SIRTs expression levels and activity, leading to a modulation of the balance between stem cell quiescence, self-renewal, and differentiation (Figure 9). Moreover, in physiological aging as well as NDs, neurons and glial cells are characterized by increased oxidative stress and several evidence suggest a key role for SIRTs in the antioxidative defense in the brain (Figure 10). However, it is still unknown whether oxidative stress can cause telomere erosion in CNS cell populations, nor if ROS-induced telomere shortening could be a causal or contributing factor to NDs. Nevertheless, SIRT1 and SIRT6 proved to play a role in telomere compaction and integrity maintenance. As we discussed, one possible explanation of ROS-dependent telomere shortening relies on the presence of GGG telomeric repeats, which are particularly sensitive ROS target sites, for the generation of 8-oxoguanine stretches, which are especially difficult to repair. The presence of unrepaired single or tandem 8-oxoguanine drastically impairs the recruitment of telomerase, thereby contributing to telomere deprotection and shortening observed in aging and NDs. To counteract this deleterious ROS-dependent effect, OGG1 activity plays an important role both at telomeres and during the NSCs differentiation. In fact, OGG1 depletion results into chronic replication stress and an accelerated telomere loss, as well as in a shift of NSCs differentiation toward the astrocytic lineage. *ogg1*^–/–^ mice differentiating neural cells spontaneously accumulate mtDNA damage coupled to a NAD^+^/NADH ratio increase, which in turn leads to SIRT1 activation. SIRT1 activation, caused by oxidizing conditions, inhibits MASH1 expression and blocks neuronal differentiation but favors a shift toward an astrocytic lineage. This evidence suggests that SIRTs, being dependent upon NAD^+^ levels, could act as metabolic sensors able to couple cellular metabolic status to a specific regulatory response. Moreover, ROS-dependent oxidation of cysteine-containing redox sensors, such as SIRT1, impairs their activity and localization, thereby regulating NSCs state and fate. Finally, the role of SIRT1 in the regulation of ROS-controlled gene expression during cellular reprogramming, corroborate the importance of this protein family in the correct NSCs lineage specification, both *in vitro* and *in vivo*.

Therefore, increased oxidative stress impact on mitochondrial physiology, by generating mtDNA damage and/or electron transport chain impairment, as well as on plasma cysteine homeostasis, thus further increasing ROS generation. Indeed, SIRTs have been shown to be involved not only in the regulation of antioxidative enzymes expression and activity, but also in the production of pro-oxidants, which, through the alteration of the NAD^+^/NADH ratio, affect SIRTs activity in a feedback loop that helps prevent the cell from entering or maintaining a state of oxidative stress.

We think that a deeper understanding of the molecular mechanism underlying the ROS-dependent regulation of SIRTs activity, as a response to cellular redox homeostasis alterations, would be of great help in the modulation of both iPSCs reprogramming and NSCs differentiation fate, as well as for a more detailed comprehension of NDs, aging, and some behavioral anomalies associated with nutrient deficiency. Moreover, a better knowledge of activation/deactivation cycles of H_2_O_2_ production and responsive redox protein systems, will help the understanding of the redox biology of neurogenesis.

## Author contributions

EM and EI: conceptualization. EM, EI, and LA: literature search. EM and CR: writing. EM, EI, CR, and LA: review and editing. All authors contributed to the manuscript and approved the submitted version.
